# Photothermal Catalytic CO_2_ Conversion: Beyond Catalysis and Photocatalysis

**DOI:** 10.1007/s41061-023-00430-z

**Published:** 2023-05-30

**Authors:** Fernando Fresno, Ana Iglesias-Juez, Juan M. Coronado

**Affiliations:** Present Address: Instituto de Catálisis y Petroleoquímica (ICP), CSIC, C/Marie Curie 2, 28049 Madrid, Spain

**Keywords:** Catalysis, Photocatalysis, Photothermal catalysis, CO_2_ conversion, Photothermal effect

## Abstract

In recent years, the combination of both thermal and photochemical contributions has provided interesting opportunities for solar upgrading of catalytic processes. Photothermal catalysis works at the interface between purely photochemical processes, which involve the direct conversion of photon energy into chemical energy, and classical thermal catalysis, in which the catalyst is activated by temperature. Thus, photothermal catalysis acts in two different ways on the energy path of the reaction. This combined catalysis, of which the fundamental principles will be reviewed here, is particularly promising for the activation of small reactive molecules at moderate temperatures compared to thermal catalysis and with higher reaction rates than those attained in photocatalysis, and it has gained a great deal of attention in the last years. Among the different applications of photothermal catalysis, CO_2_ conversion is probably the most studied, although reaction mechanisms and photonic-thermal synergy pathways are still quite unclear and, from the reaction route point of view, it can be said that photothermal-catalytic CO_2_ reduction processes are still in their infancy. This article intends to provide an overview of the principles underpinning photothermal catalysis and its application to the conversion of CO_2_ into useful molecules, with application essentially as fuels but also as chemical building blocks. The most relevant specific cases published to date will be also reviewed from the viewpoint of selectivity towards the most frequent target products.

## Introduction

As is widely recognized, catalysis is key for the development of sustainable chemical processes, which in turn are inevitably necessary in order to attain the Sustainable Development Goals related to water, energy, industry and the environment. Indeed, catalysis allows all the principles of green chemistry to be fulfilled [1], since (1) it helps obtaining fuels alternative to fossil ones and less polluting; (2) it contributes to saving raw materials and energy by optimizing processes; (3) it facilitates the use of renewable raw materials; and (4) it favours the design of clean processes, respectful of the environment. This refers not only to the increase in the reaction rate that defines the action of a catalyst, but also to the selectivity that can be achieved in certain processes by using the right catalyst. Thus, a selective catalytic process (1) minimizes the formation of by-products and residues; (2) maximizes the incorporation of the reactants to the desired product, which results in atomic efficiency; (3) reduces or eliminates the need for derivatization; and (4) reduces the use of auxiliary substances. For these reasons, catalysis is ubiquitous in industry and, in fact, most processes in the chemical industry (approximately 90%) require the use of a catalyst in at least one step of the process.

In addition to green chemical processes, it is clear that sustainable development requires that renewable energy sources, solar *par excellence*, increasingly and progressively replace fossil ones. In this sense, catalysis may have a two-way relationship with renewable energy. On the one hand, it can be used for the conversion of primary energy sources, for example by converting biomass into fuels [2]. On the other hand, a renewable energy source can be used at the same time to activate catalytic processes, and in this case photocatalysis would be the archetypical example [3], although thermal catalytic processes can also be driven by sunlight if they are coupled to solar concentrating systems [4].

Besides, sustainable development necessarily requires the reduction of CO_2_ emissions and the stabilization of its concentration in the atmosphere, as reflected by the International Energy Agency in its projections [5]. To this end, converting waste CO_2_ into a renewable raw material can play an important role, thus complying with one of the fundamental pillars of green chemistry, namely the use of renewable raw materials, in line with the concept of a circular economy. Today, CO_2_ is used as a raw material in different chemical industrial processes and to a lesser extent in other applications. The latter include, for instance, enhanced oil recovery, its use as a supercritical solvent, the carbonation of beverages, or as a protective atmosphere for packaging. Regarding its use after chemical conversion, the cultivation of microalgae to obtain biomass for energy purposes, or different synthetic processes of the chemical and pharmaceutical industry, can be mentioned. Nevertheless, the sum of all these uses represents only a small fraction of the anthropogenic emissions of this gas into the atmosphere [6]. Among the possible uses of CO_2_, however, the one that is furthest from the market is its conversion into fuels [7]. Thus, achieving a viable use of CO_2_ for its incorporation into the circular economy as a raw material is in fact considered one of the *grails* of modern chemistry [8]. Catalysis plays a fundamental role here due to the chemical inertness of this molecule, and the catalytic conversion of CO_2_ is considered a key technology to fully introduce renewable energy sources in the chemical industry, considering that most of the energy consumption of this type of industries is in the form of fuels rather than electricity [9]. Recalling the two-way relationship between renewables and catalysis, solar-powered catalysis would then offer the possibility of storing solar energy and using CO_2_ in the same process, providing an alternative to fossil feedstock both as energy source and as a raw material.

**Fig. 1:**
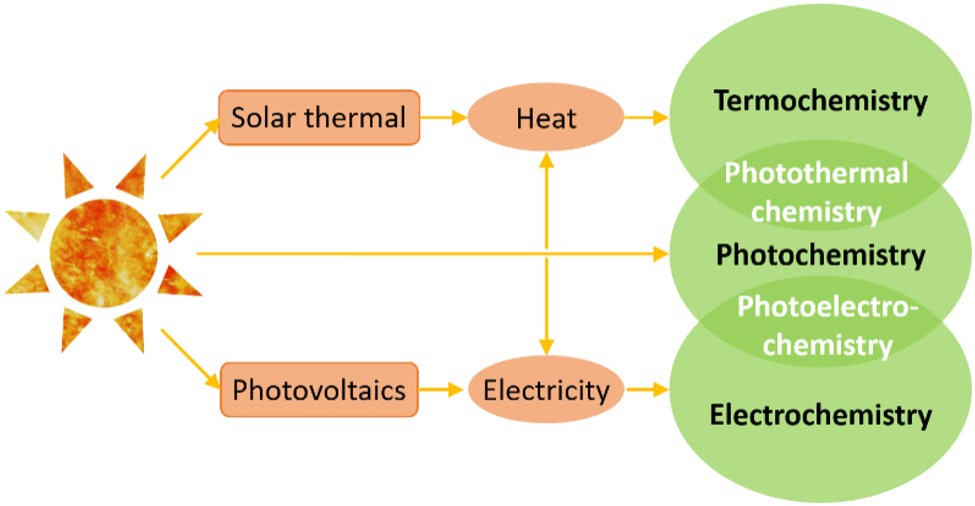
Schematic representation of the direct and indirect uses of solar energy to drive chemical reactions. Photothermal chemistry works at the interface of photochemistry and thermochemistry. Adapted with permission from Ref. [11]. Copyright The Royal Society of Chemistry 2019

As its name indicates, *photothermal catalysis* works, as illustrated in Fig. 1, at the interface between purely photochemical processes, which involve the direct conversion of photon energy into chemical energy, and classical thermal catalysis, in which the catalyst is activated by temperature. Thus, photothermal catalysis acts in a double way on the energy path of the reaction: on the one hand, reducing the activation energy through photocatalysis and, on the other, increasing the probability of overcoming this barrier by providing extra thermal energy. This *combined catalysis*, of which the fundamental principles will be reviewed in the following section, is particularly promising for the activation of rather unreactive molecules like CO_2_ at moderate temperatures compared to thermal catalysis and with higher reaction rates than those attained in photocatalysis, and it has gained a great deal of attention in the last years [10–14]. Among the different applications of photothermal catalysis [15], CO_2_ conversion is probably the most studied, and most of the published works deal with the production of CO or CH_4_, as will be reviewed below, while other products like methanol, ethanol and C_2+_ hydrocarbons are less frequently reported [13, 14].

This article intends to provide an overview of the principles underpinning photothermal catalysis and its application to the conversion of CO_2_ into useful molecules, with use essentially as fuels but also as chemical building blocks. The most relevant specific cases published to date will be also reviewed from the viewpoint of selectivity towards the most frequent target products.

## Principles of Photo-Thermal Catalysis

### Photonic and Thermal Synergies

The fundamental principle of heterogeneous photocatalysis is based on the formation of electron–hole pairs upon irradiation of a semiconductor with photon energy equal to or greater than its bandgap. These photogenerated charges may live long enough to migrate to the surface of the solid and react there with adsorbed species, catalysing oxidation–reduction reactions. The physico-chemical properties of the solid affect the photocatalytic process in several ways: the bandgap of the photocatalyst determines the wavelength of light that can be used to activate it; charge separation is facilitated in high dielectric constant semiconductors because the carriers are shielded by the lattice; the ability of charge carriers to migrate towards the surface depends on structural (crystallinity, defects…) and electronic (involved orbitals…) factors of the semiconductor; the adsorption of reactants and desorption of products depend on the morphology (surface area, porosity) and surface chemistry of the semiconductor; and last but not least, the energies (reduction potentials) of the conduction and valence bands determine the reduction and oxidation half-reactions that can occur.

For its part, thermal heterogeneous catalysis is based in many cases on the reactivity of low-coordination sites at the surface of solids, which provides a lower energy pathway for molecules to rearrange their bonds in the breaking and reforming that is required for a chemical reaction [16]. Even if activation energy of the reaction is decreased by the catalysis, there is a temperature dependence of the reaction outcome that depends not only on the thermodynamics but also on the specific reactivity of the catalyst surface and on the heat of adsorption and desorption of reactants and products, which altogether results in that, very often, catalytic reactions show a turnover in reaction rate at a certain temperature. The working temperature of most industrially relevant heterogeneous catalytic reactions is thus of several hundred degrees Celsius (apart from more or less high pressures), which not only redounds in the cost and environmental impact of the process, but can also lead to undesired outcomes such as catalyst coking or sintering.

From the thermodynamic point of view, the main difference between photo- and thermal catalysis is that the latter is restricted to the acceleration of spontaneous (i.e. Gibbs energy *downhill*) reactions, which implies that, depending on the process, temperature and pressure may need to be increased so that this condition is met. On the other hand, photocatalysis challenges the definition of catalysis in the sense that it can also promote *uphill* reactions (as is the case of water splitting and CO_2_ reduction with water), which provides photocatalysis with the ability of storing photon (solar) energy into chemical bonds. Strictly speaking, these processes should be referred to as photosynthetic rather than photocatalytic [17], but they are commonly included under the umbrella of the latter.

**Fig. 2:**
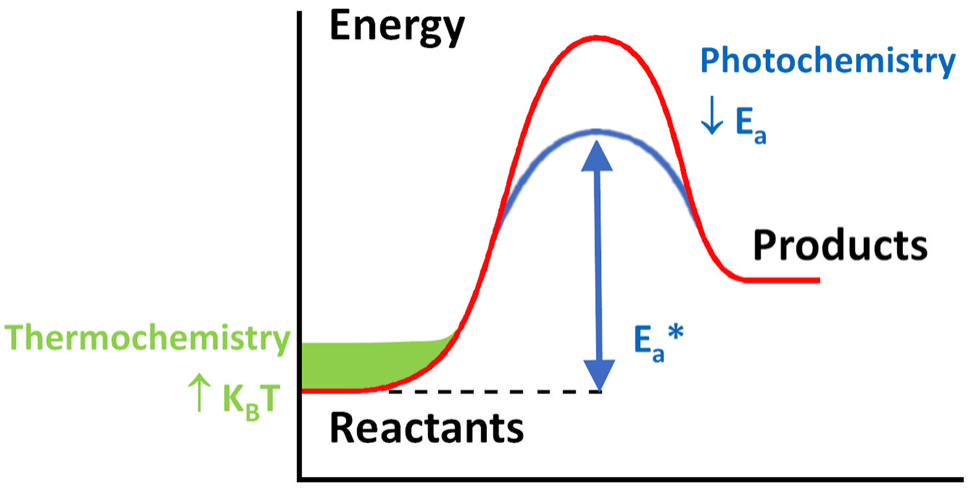
Effect of the photochemical and thermochemical contributions of photothermal catalysis to the energy path of an endothermic reaction. Adapted with permission from Ref. [11]. Copyright The Royal Society of Chemistry 2019

The photothermal effect has been known for a long time [18] and essentially consists in the conversion of radiant energy absorbed by a substance into heat, with a consequent local increase of temperature in this substance. This apparently simple principle opens up a vast range of applications ranging from tumour diagnosis and treatment [19] to passive energy applications such as reducing heat loss in building structures [20], through materials characterization [21], bacteria inactivation [22] drug delivery [23] or catalysis. In the latter, as outlined in the previous section, photothermal catalysis opens up the opportunity of increasing the conversion of photocatalytic reactions while keeping process temperatures below those necessary for the corresponding thermo-catalytic processes. This occurs by means of a two-way activation path. Essentially, it is not a mere superposition of photochemical and thermal processes, but it arises from the synergy of different physical phenomena [11]: on the one hand, the generation of electron–hole pairs upon semiconductor excitation with light and, on the other hand, the local heating produced by plasmonic or non-plasmonic nanoheaters, of which the specific mechanisms will be outlined below. Figure 2 represents the contribution of both components to the energy path of a chemical reaction. The photochemical contribution increases the reaction rate by lowering the activation energy of one or several reaction steps, by either charge transport from the bulk to the surface of the semiconductor, which induces the formation of surface radicals (e.g., ·OH) or other reactive species such as reduced metal ions, or energy transfer in the form of a vibrational mode to the transition state, which causes elongation of chemical bonds. In turn, the thermochemical component enhances the reaction rate by increasing the local temperature at the active sites. In a reaction energy path (Fig. 2), this means a growth of the probability of overcoming the energy barrier by means of a displacement of the Boltzmann distribution to higher energy. The effect on reaction kinetics is therefore equivalent to that explained above for heterogeneous catalysis.

Focusing on CO_2_ conversion, thermal activation provides higher reaction rates (reaching the range of mmol g_cat_^−1^ h^−1^) with respect to photocatalysis; on the other hand, with respect to thermal catalysis, lower *macroscopic* temperature can be used by introducing photochemical activation and, in addition, different reaction mechanisms can occur leading to different selectivities. Lowering the process temperature also disfavours the formation of coke that usually leads to deactivation of the catalysts.

This dual activation concept can be applied to different reactions of interest for CO_2_ conversion, such as dry reforming of methane (Eq. 1), reverse water gas shift (Eq. 2), the Sabatier reaction (Eq. 3) or methanol synthesis (Eq. 4), together with the various pathways that result from the reaction of CO_2_ with water [24]. Among those, we will pay special attention to those involving the reaction of CO_2_ with H_2_.

1 $${\text{CO}}_{{2}} + {\text{CH}}_{{4}} \rightleftarrows {\text{2CO}} + {\text{2 H}}_{{2}}$$

2 $${\text{CO}}_{{2}} + {\text{H}}_{{2}} \rightleftarrows {\text{CO}} + {\text{H}}_{{2}} {\text{O}}$$

3 $${\text{CO}}_{{2}} + {\text{4H}}_{{2}} \rightleftarrows {\text{CH}}_{{4}} + {\text{2H}}_{{2}} {\text{O}}$$

4 $${\text{CO}}_{{2}} + {\text{3H}}_{{2}} \rightleftarrows {\text{CH}}_{{3}} {\text{OH}} + {\text{H}}_{{2}} {\text{O}}$$

### Plasmonic Photothermal Catalysis

Resonant optical excitation of surface plasmons can be used to produce catalytic enhancement through associated photochemical and thermal contributions (photothermal effects) derived from the non-radiative plasmon decay. Let us comment first about some fundamental concepts.

*Plasmons* define the collective oscillations of free electron gas density and can be classically described as an oscillation of the electron density with respect to the fixed positive ions inside a *plasmonic* particle. This oscillatory charge motion inside the material can be induced by an external incident wave. Thus, the light causes a coherent motion of the metal conduction electrons with the external electric field oscillations. The electron cloud displacement relative to its equilibrium position induces a coulombic attraction force between the nuclei and the electrons. This restoring force produces the electron cloud fluctuation so that the system behaves like a harmonic oscillator. The electric charge density fluctuation will propagate through the entire solid in a quantized manner where each quantum of oscillation is a *plasmon* [25]. When resonant conditions occur, both the incident electromagnetic field and the frequency of the electrons are in phase, thus maximizing the electric field on the surface of the plasmonic particle. Different factors, such as density of electrons, effective electron mass, size and shape both of the charge distribution and of the particle, etc. determine the vibration frequency [26]. If the perturbation is restricted to the surface of the metal it is called a *surface plasmon*. In surface plasmons, the interface between the metal and the surrounding medium modifies the properties with respect to those of volume plasmons. *Localized surface plasmons* (LSP) result from the confinement of a surface plasmon in a nanoparticle (NP) of size similar to or smaller than the wavelength of the light used for plasmon excitation.

The excitation of surface plasmons of metallic nanostructures by light is known as *localized surface plasmon resonance* (LSPR). LSPR has important effects on the nanostructures: their electric fields near the surface are greatly increased, which derives in a larger optical absorption cross section resulting in an antenna-like effect that boosts light absorption around the NPs. This optical absorption has a maximum at the plasmon resonant frequency, so that the capacity of the NPs to efficiently collect light is often limited to a few resonant frequencies, although there are exceptions in which plasmonic structures can efficiently capture light over a broadband spectrum [27]. Metallic NPs and certain semiconductor nanostructures (including metal oxides, chalcogenides, nitrides, silicon…) can present LSPR that after excitation can lead to different types of associated processes that will contribute to the photothermal effects [28]. Lately, graphene has also been shown to activate surface plasmons in the range of terahertz to mid-infrared frequencies [29].

Following optical activation, the LSPR energy de-excitation or *damping* takes place, either in a radiative way by photon re-emission or by non-radiative pathways. These processes take place over a period in the order of femtoseconds to picoseconds. The radiative decay is due to direct photon emission by coherent electron oscillation. As the size of the NP increases, the radiative decay of the plasmon is more significant. For larger NPs, the radiative decay component is the main reason of plasmon resonance broadening and dipole strength weakening. In contrast, decreasing the size of the NP lets the nonradiative component dominate the plasmon decay.

Non-radiative pathways include *Landau damping* and *resistive loss* and occur through different types of processes involving electron–electron and other inelastic collisions that take place during the oscillatory electron cloud motion. Landau damping (electronic scattering with the surface of the plasmonic structure) gives rise to electron promotion (e^−^/h^+^ pair generation) via direct interband or phonon/geometry-assisted intraband transitions by energy transfer from the plasmon quantum (timescale of femtoseconds, 1–100 fs) [30]. The intraband transition happens in the conduction band and the interband transition can occur between other bands, such as the d-band, and the conduction band [31, 32]. As the energy of these excited carriers is much larger than the thermally excited electron–hole pairs at ambient temperature, they are called *hot carriers*. The formation of hot electrons is usually generated due to intraband transitions, whereby, they are able to transverse Schottky barriers. On the other hand, interband transitions typically afford energetic (hot) holes, where the excited electrons are not able to overcome Schottky barriers. In short, intraband transitions generates hot electrons, whereas interband transition produces hot holes [33]. Electron–electron collisions can also produce a rapid non-equilibrium heating increasing the temperature. In the case of resistive loss, single carriers, electrons or holes, of the plasmon quasi-particle are ejected out of the phase-coherent collective plasma oscillation through electron–electron or electron–phonon scattering.

Hot carriers originating from LSPR decay are distinguished from other types of electronic excitations in that they are not in thermal equilibrium with the material and therefore they are described by the Fermi–Dirac distribution with an elevated effective temperature [34]. These hot charges (h^+^/e^−^) can recombine (relaxation) or escape from the plasmonic NPs and induce further chemical reactions. The relaxation takes place through scattering processes that redistribute the charge carriers and thermalize them in the order of one to hundreds of picoseconds generating long-lived hot carrier distribution. This thermalization process occurs by electron–electron scattering that increases surface temperature locally, by electron–phonon scattering increasing the temperature of the metal lattice via phonon modes activation and then by phonon–phonon scattering that dissipates the thermal energy through the NP lattice. Finally, cooling process and energy transfer to the substrate happen by phonon and geometry-assisted scattering pathways over a longer time scale (hundreds of picoseconds to tens of nanoseconds), driving the relaxation of the hot carrier distribution to the equilibrium.

As mentioned above, the hot carriers (electrons or holes possessing sufficient energy) can also migrate from the NPs by direct or indirect transfer to molecules adsorbed on their surface or, in the case of heterostructured systems (supported metal NPs), into the semiconductor support. These four injection routes, i.e. direct or indirect transfer to either adsorbed molecules or the support, can induce further chemical reactions with an important chemical effect on catalysis. Electron transfer processes have received more attention than hot-hole-driven ones due to the shorter lifetime of hot holes (fs to ps) compared to hot electrons, which makes it more difficult to drive redox reactions as the time scale of chemical reactions is usually in the range of ms or sub-ms. In addition, their short life makes their experimental investigation more difficult [35], and the information is obtained indirectly by monitoring the state occupation in the semiconductor support or by using hot hole scavengers as sacrificial reagents. Recently, ultrafast spectroscopy has been employed to study the relaxation and transfer processes of hot holes [36], showing their great potential. Indeed, the potential of plasmon-generated hot holes in chemical conversions has been proven effective in driving many photochemical reactions, including organic transformations, metal etching, oxide deposition and oxygen evolution. To exploit this pathway for highly efficient chemical conversions it is necessary to effectively separate and collect these hot holes by constructing adequate interfaces with the semiconductor or the adsorbed molecules with a suitable energy band/level structure [35].

Direct electron transfer to the adsorbate proceeds through direct generation of hot electrons into hybridized states between the adsorbed molecules and metal NPs, known as chemical interface damping (CID). A strong interaction between the adsorbate and the NP is required for surface orbital hybridization, which is not usual in plasmonic particles, and then the generated acceptor state has to be resonant with the plasmon energy to allow direct injection. Indirect electron transfer to the adsorbate is a two-step process where hot electrons are first generated within the metal nanoparticle and subsequently transferred to the lowest unoccupied molecular orbital (LUMO) of the adsorbed species. In turn, as it happens with adsorbates, direct or indirect electron transfer to the semiconductor conduction band is possible if the energy is higher than the Schottky barrier formed at the metal-NP/semiconductor interface. These injection pathways occur in the femtosecond range. Direct injection processes take place in a single step, being faster (fs order) than the two-step indirect transfer processes. The former are also more efficient because they avoid the energy loss of hot carriers associated to electron–electron and electron–phonon scattering within the metal NP.

These hot charge transfer processes favour the spatial separation of photo induced electron–hole pairs once electrons are promoted to the adsorbate or semiconductor, preventing charge carrier recombination within the metal and thus extending their lifetime. Also, the redistribution of charge induces further chemical reactions and open new activation routes and possibilities of selectivity control. As noted above, generated plasmons have also thermal effects as they can inelastically decay through a plasmon–phonon interaction, increasing substantially the local temperature. Equally, the induced hot carriers can also decay to convert energy to heat. Therefore, plasmonic heating also plays a crucial role in the photocatalytic activity enhancement but the use of plasmon heating to perform chemical reactions is similar to externally heating the system and does not offer other pathways to control product selectivity. Therefore, in the case of plasmonic materials, the photo-thermal effect arises from the combination of both thermal and photochemical contributions of non-radiative plasmon decay.

### Non-plasmonic Photothermal Catalysis

In addition to plasmon activation which, as mentioned in the previous section, takes place at a relatively narrow interval of wavelength, generally in the UV–visible region for metal particles, photothermal catalysis can also take advantage of a wider portion of the solar spectrum by using materials with broad-range absorption. For example, photon absorption by dark coloured materials, which can be excited even by infrared radiation, leads to heat generation caused by non-radiative relaxation processes that transfer photon energy to the lattice phonons [11, 37]. In metallic particles, non-radiative decay following irradiation can take place by both intraband (e.g. from the conduction band to d-levels) and interband hot carrier relaxation. In semiconductors, heat generation arises from Auger recombination, in which the energy given off by electron–hole recombination is absorbed by a third electron excited to higher energy states within the same band. Electron–hole pair recombination can also occur through Shockley–Read–Hall processes. This last non-radiative mechanism is also known as trap-assisted recombination and it implies an initial fast relaxation of the electron to a localized energy state in the bandgap, before fully decaying to the ground state. This relaxation route is important for metal oxides which often present defects such as oxygen vacancies that can act as electron traps [11]. These events are very fast, with typical decay times in the 10–100 picoseconds for Auger recombination and about tens of nanoseconds for slower Shockley–Read–Hall recombination.

Heat capacity and thermal conductivity are also very relevant properties to establish the final efficiency of photothermal catalysts because they influence the temperature achieved locally at the active sites. Convective thermal transport can be important for large scale reactors. On the other hand, selective spectral absorbent coatings, which are characterized by efficient absorption in the visible and low radiative losses in the IR, has been indirectly exploited in photothermal catalysis to achieve efficient heating by irradiation [38]. However, in most cases thermal conductivity is the predominant mechanism of heat dissipation in photothermal catalysis. In the case of metals, electrons are the main thermal carriers, while in other materials such as oxides, thermal conductivity is driven by phonon scattering [11]. Although these are mainly intrinsic properties of the solid phases, there are some possibilities of modulation by changing material morphologies because lattice imperfections and grain boundaries increase the phonon scattering. For example, reducing the particles size contributes to diminished thermal conductivity and, eventually, can increase the temperature achieved by the illuminated material. An additional decrease in thermal conductivity can be induced by confinement effects in nanoparticles. Furthermore, the multiphasic character of photothermal catalysts provides an opportunity to independently adjust the thermal response of each component of the catalysts to maximize the energy harvesting. Thus, ideally, the optical absorber component performing the photothermal conversion should have high thermal conductivity to efficiently deliver the energy to the active site, while support with thermal insulation characteristics may be preferred for avoid subsequent heat dissipation.

**Fig. 3:**
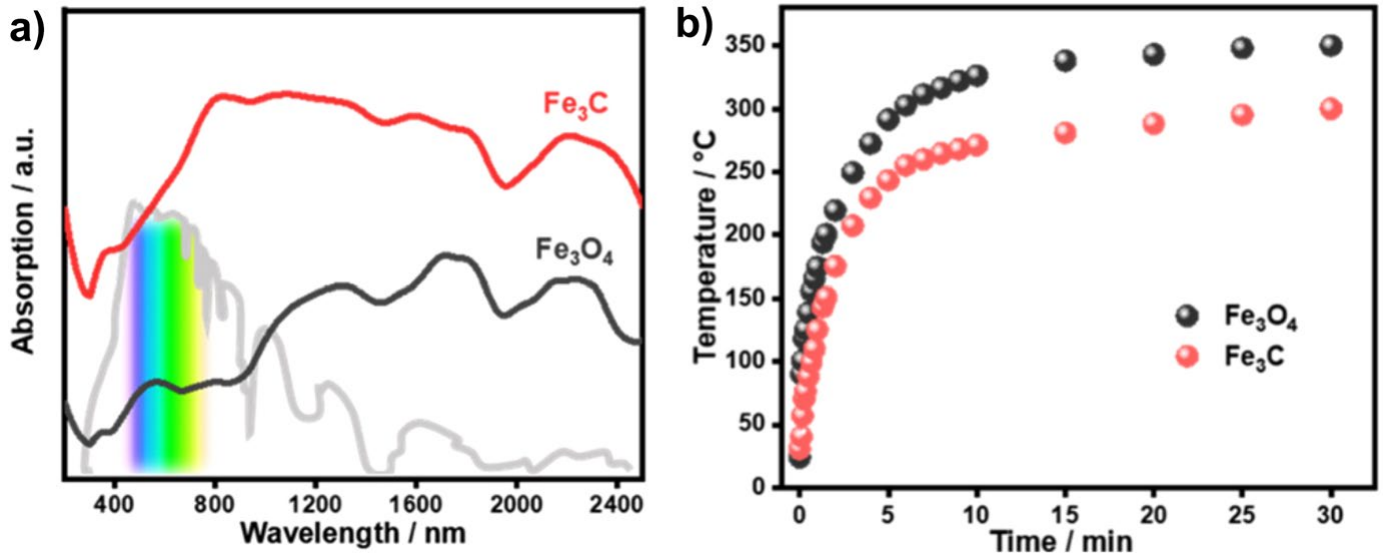
**a** UV–Vis–NIR absorption spectra of Fe_3_O_4_ and Fe_3_C photothermal catalysts and **b** time evolution of the temperature profiles of Fe_3_O_4_ and Fe_3_C under illumination with a 300 W Xe lamp. Reproduced with permission from Ref. [39]. Copyright American Chemical Society 2020

Compositions of non-plasmonic photothermal catalysts are rather varied, comprising supported metal particles, metal oxides, carbides, sulphides and MXenes, among other compounds including metal organic frameworks and other carbon-based materials such as graphene or carbon nanotubes. Transition elements and in particular group VIII metals have good photothermal conversion efficiency and they have been often applied with this purpose because they are abundant and low-cost elements. In the context of CO_2_ hydrogenation, remarkable results in hydrocarbon production have been obtained with catalyst containing different proportions of Fe_3_O_4_, Fe and Fe_3_C phases [39]. Iron carbide is highly selective towards methane production, and while a significant yield of C_2+_ is obtained with the mixed phase containing metallic Fe and Fe_3_C. Under illumination with a 300-W Xe lamp (2.05 W cm^−2^), Fe_3_O_4_ reaches a temperature as high as 350 °C in about 15 min, while in the same conditions Fe_3_C only reaches about 250 °C (see Fig. 3). This illustrates the influence of the optical characteristics of the material on the efficiency of light-to-heat conversion, since Fe_3_O_4_ presents higher absorbance in a broad wavelength range. Similarly, Ni/Nb_2_C can exceed 300 °C under illumination with a Xe lamp (15 suns), achieving a high yield of methane [40]. Silicon nanowires can be used to increase the conversion of CO_2_ to CO by In_2_O_3–x_(OH)_y_ nanoparticles under solar light illumination due to their broad absorption and efficient photothermal conversion [41]. Another example of photothermal catalysts without plasmonic components for CO_2_ conversion is the mixture of α-MoC and β-Mo_2_C phases, which has shown a 45% increase in methane production under photothermal conditions relative to purely thermal activation [42]. Similarly, Co–Cu–Mn tricomponent oxides, containing both oxide and metallic particles, show an enhancement of methane production under photothermal conditions [43]. Alternatively, narrow bandgap semiconductors with absorption in the IR range can be used as photothermal catalysts. In this way, CuS has been successfully combined with the UV-active TiO_2_ to obtain an efficient broad-absorption catalyst for the reduction of CO_2_ to CO [44].

**Fig. 4:**
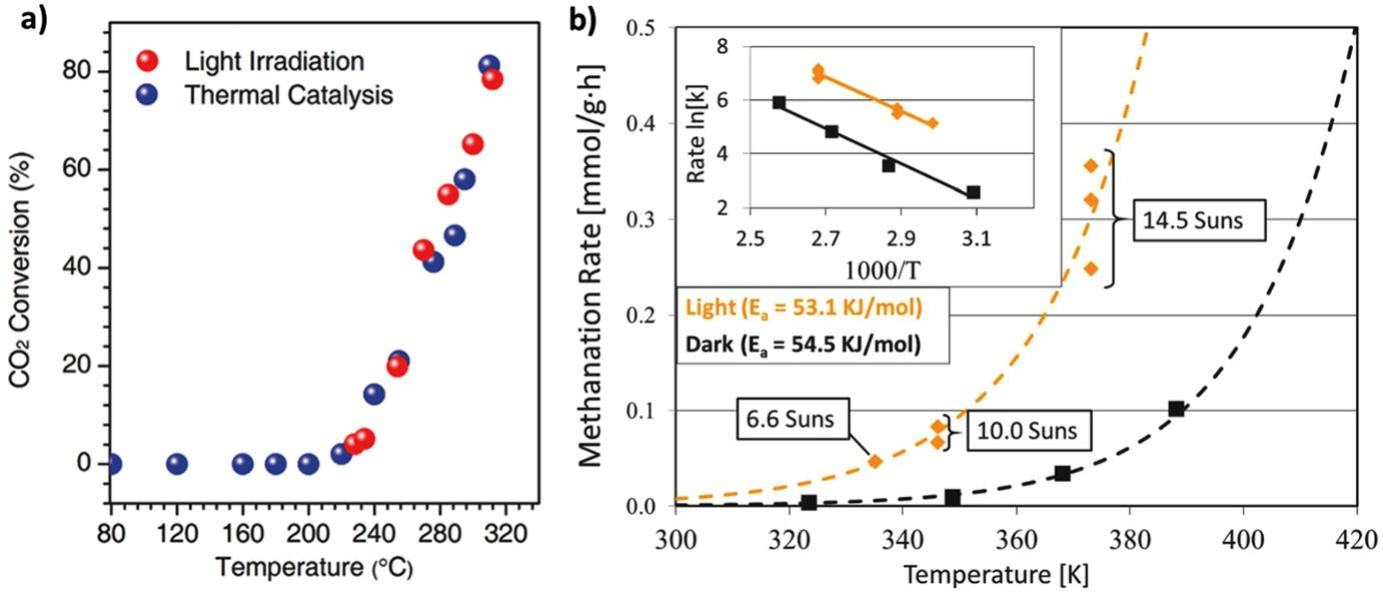
**a** Comparison of CO_2_ conversion during hydrogenation using CoFe/Al_2_O_3_ catalyst under photothermal heating (UV–Vis irradiation) and direct thermal heating (no UV–Vis irradiation) Reproduced with permission from Ref. [45]. Copyright Wiley 2018. **b** Rate of the photothermal methanation over Ru supported on Si nanowires plotted as a function of temperature in the dark (black) and under solar-simulated irradiation (yellow). The inset shows these methanation rates on a plot of ln(*k*) versus 1000/T used to calculate the activation energy. Reproduced with permission from Ref. [46]. Copyright Wiley 2020

In non-plasmonic systems, the main effect of visible/infrared (IR) irradiation appears to be the selective heating of the catalysts. This effect can have advantages in terms of energy efficiency, due to the delivery of the heat specifically to the active sites where is needed to activate the reaction of interest. However, additional synergies due to electronic transferences such as those described for plasmonic-semiconductor systems are not usually observed. In this regard, the catalytic performance can be nearly identical under conventional or illumination-mediated heating. This was confirmed for catalysts based on Co and Fe alloys supported on alumina during CO_2_ hydrogenation. In Fig. 4a, CO_2_ conversion is plotted against reaction temperature under UV–Vis irradiation (i.e., heating by photon-to-heat conversion) and under direct external heating, indicating the equivalence of both activation procedures [45]. Nevertheless, in some cases the effect of irradiation clearly improves the results obtained using only thermal activation. This is the case of the methanation of CO_2_ over Ru supported on Si nanowires (black silicon), which is illustrated in Fig. 4b. For this system, the light-off curve is shifted towards lower temperatures (ca 30 °C) upon illumination [46]. However, the fact that activation energy determined in the dark and under illumination is almost the same (about 54 kJ mol^−1^) suggests that the mechanism for the two routes is similar. This points to a local increase in temperature upon illumination, which is difficult to determine with conventional methods, while the contribution of synergic effects between heat and light activation also appears to play a role on the activation of hydrogen.

In summary, non-plasmonic systems provide a feasible route to use solar light to generate heat to efficiently activate catalytic processes at active site level. Additional cooperative effects between photonic and thermal activation are not unambiguously observed. However, these cooperative effects cannot be completely discarded, and they could provide a via for improving the performance of these catalysts.

## Photothermal CO_2_ Conversion: Beyond Catalysis and Photocatalysis

### CO_2_ + H_2_O: Artificial Photosynthesis

**Fig. 5:**
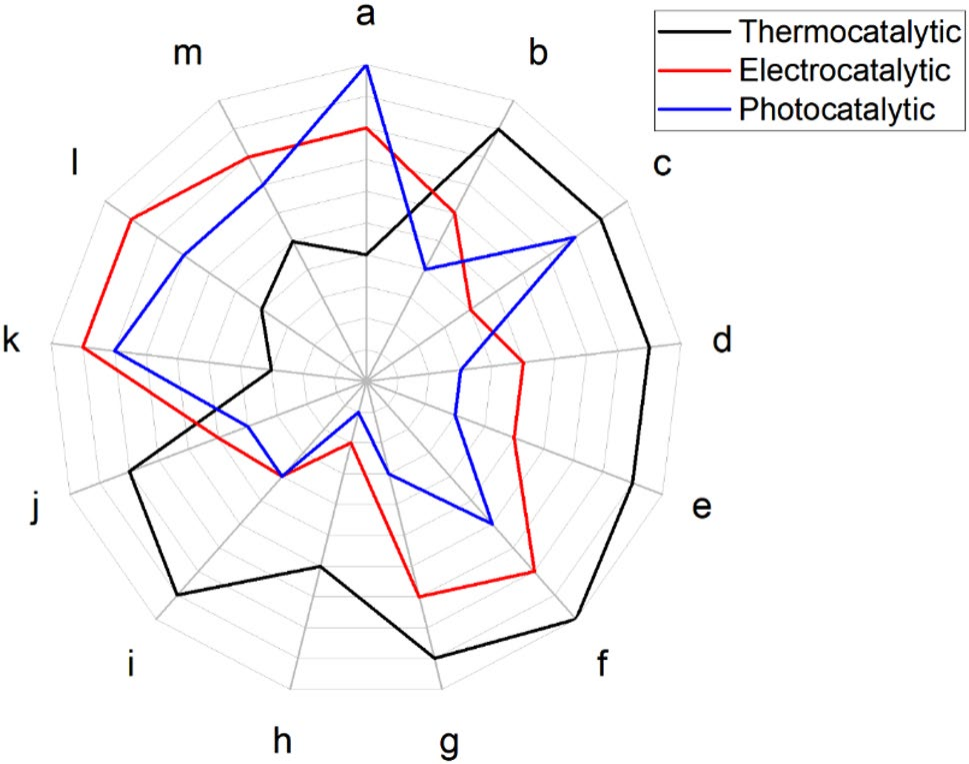
Comparison of different catalytic pathways for CO_2_ conversion according to: **a** ideal predicted energy efficiency of these technologies; **b** potential breakdown of commercial barriers; **c** relative scalability; **d** compared activity of state-of-the-art catalysts; **e** attained shifts to longer chain hydrocarbons; **f** selectivity to methane; **g** selectivity to olefins; **h** selectivity to long-chain hydrocarbons; **i** currently reported catalyst stability; **j** current mechanism knowledge; **k** recent catalyst development; **l** emergence of high-impact breakthroughs; **m** catalyst synthesis easiness. Adapted with permission from Ref. [48]. Copyright The Royal Society of Chemistry 2019

Artificial photosynthesis (AP), i.e. converting CO_2_ into reduced products using water as electron donor by means of light-activated processes, is probably one of the most elusive challenges that science faces today. Photosynthetic organisms have performed this complex task for millions of years and, although their mechanisms are mostly known, the efficiency, selectivity and stability issues that can lead to a practical device mimicking this ability in an artificial way have not been addressed yet, even if intensive research is being carried out. Among the different approaches to AP, heterogeneous photocatalysis of the CO_2_ + H_2_O reaction involves a direct solar-to-chemical energy conversion, and thus exhibits relatively high theoretical efficiencies. This fact, together with environmental and social aspects, makes it especially promising [47], although the practical efficiencies reported to date are still low and its technological development is at the early-research/proof-of-concept stage. The scientific relevance (measured as number of publications) of photocatalytic CO_2_ reduction has grown exponentially in the last 10–15 years, in spite of which there are still unknown critical aspects of the reaction that may be hindering its further development. Figure 5 depicts a comparison of photocatalysis with two other technologies for CO_2_ reduction: thermal catalysis and electrocatalysis [48]. As shown by this analysis, photocatalysis is more relevant in figures of merit related to its potentiality, like theoretical efficiency and scalability, in addition to the already mentioned scientific relevance, while it is less developed in other figures more related to its state of development such as catalyst stability, control of selectivity and mechanistic knowledge. Some of the identified bottlenecks that may hinder the development of photocatalytic CO_2_ reduction can be summarized as follows: (1) CO_2_ molecule activation; (2) the oxidation of water for electron counterbalance; (3) the competing parallel reduction of H_2_O to H_2_; and (4) catalyst stability [24].

Regarding CO_2_ activation, the extra thermal energy provided to the reactants in photothermal catalysis, as outlined in Sect. 2.1, can increase the probability of activating CO_2_ and overtaking the energy barrier [11]. In addition, density-functional theory (DFT) studies have shown that oxygen deficiencies created thermally on reducible oxides like MnO_x_ may enhance the reactive adsorption of CO_2_ to yield a CO molecule [49]. On the other hand, the oxidation of water is a critical aspect in photocatalytic artificial photosynthesis since it is a complex, kinetically hindered reaction. It is rare, however, to find the use of sacrificial hole acceptors in CO_2_ reduction, although the use of sulphites, alcohols or amines have been reported [50]. In pure photocatalytic processes, there is a wide dispersion of results in terms of oxygen formation, with articles reporting non-stoichiometric oxygen evolution or even its complete absence, for which several factors, like incomplete water oxidation, re-oxidation of products by produced oxygen or the behaviour of carbonates as hole scavengers, have been invoked [24]. In photothermal CO_2_ + H_2_O catalysis (Table 1), it has been proposed that hydrogen coming from photocatalytic water splitting (H_2_O → H_2_ + O_2_) reduces CO_2_ to methane with high selectivity, but without oxygen evolution reported [51, 52]. Also, the formation of formic acid has been proposed to occur via a similar pathway, without a reported electron balance, in a purely photothermal system based on plasmonic Pt-Au/SiO_2_ materials [53]. Specific studies on the improvement of water oxidation by the photothermal effect have been, to the best of our knowledge, limited to photoelectrochemical or photothermal electrocatalytic systems [54].

**Table 1:** Some published results of photothermal CO_2_ + H_2_O catalysis

Catalyst	Temperature (K)	Light	Main product	Formation rate (µmol h^−1^)	References
Au-Ru/TiO_2_	423	UV–Vis (Hg)	CH_4_	0.81	[51]
TiO_2-x_	393	Vis-UV (Xe)	CO	0.34	[52]
Pt/TiO_2-x_	393	Vis-UV (Xe)	CH_4_	0.3412	[52]
Pt-Au/SiO_2_	–*	Vis-UV (Xe)	HCOOH	0.324	[53]
Pt-Au/SiO_2_	–*	NIR (808 nm laser)	HCOOH	0.918	[53]
Pt-Au/SiO_2_	–*	Solar	HCOOH	0.594	[53]

*No external heating applied

The formation of hydrogen by means of water reduction, competing with CO_2_ reduction for conduction band electrons, is a common outcome of the photocatalytic CO_2_ + H_2_O reaction and, as indicated above, has been invoked to account for the conversion of CO_2_ into reduced products by a two-step reaction involving water splitting and hydrogenation [13, 24]. On the other hand, hydrogen evolution can be seen as a drawback since it decreases actual CO_2_ reduction by photoexcited electrons, and many efforts have been devoted to drive this competition towards CO_2_ reduction [55, 56]. However, in connection with the two-step reaction mentioned above, there is the question whether it is wise to concentrate efforts on suppressing the more favourable hydrogen evolution, or if it would be more desirable to use photocatalysis to produce green hydrogen and then conduct CO_2_ hydrogenation [57]. Actually, there is a considerably larger number of works dealing with the latter in photothermal catalysis and, in those studying direct reduction with water, conversions remain lower and selectivities more restricted in comparison with the hydrogenation reactions reviewed in the following sections [13].

Catalyst deactivation is also among the main problems of photocatalytic artificial photosynthesis, and its mechanism has not been fully understood yet in order to avoid it. Indeed, most reported catalysts have been shown to remain active for just a few hours [58]. Some of the factors that have been put forward to explain catalyst deactivation include catalyst poisoning by blocking of the active site by intermediates; slow desorption of products or intermediates; agglomeration or sintering of cocatalysts; and exhaustion of active sites along the reaction [24].

### Methane Production: Sabatier Reaction

Methane can be obtained from direct hydrogenation of CO_2_ in the so-called Sabatier reaction, also referred to as the CO_2_ methanation process (Eq. 3), with ΔH _298 K_ = −165.0 kJ mol^−1^. This way of obtaining synthetic natural gas plays a pivotal role in the energy transformation system as it contributes to reducing atmospheric CO_2_ concentrations and provides a valuable gas fuel compatible with current technologies that can be directly injected in existing pipeline networks or storage infrastructures [59, 60].

The most widely accepted reaction mechanism of the overall exothermic reaction is the combination of the endothermic reverse water–gas shift reaction (RWGS, Eq. 2, Δ*H*_298K_ = 41 kJ mol^−1^) and the exothermic CO methanation (Eq. 5) [61, 62]. Thus, high temperatures promote CO evolution, resulting in the decrease of CH_4_ selectivity.

5 $${\text{CO}}\, + \,{\text{3H}}_{{2}} \to {\text{CH}}_{{4}} \, + \,{\text{H}}_{{2}} {\text{O}}\Delta {\text{H}}_{{{298} {\text{K}}}} \, = - {2}0{6}\; {\text{kJ}}\;{\text{mol}}^{{ - {1}}}$$

Ru-, Rh-, Ni- or Co-based catalysts supported on different oxides have been extensively studied [59, 63–65]. However, Ni-based catalysts remain the most widely explored materials mainly due to their low cost, despite the highest turnover numbers achieved with Ru-based catalysts [63, 66]. The nature of the support also plays a crucial role for high CO_2_ methanation activities and the most suitable supports explored have been Al_2_O_3_, TiO_2_, CeO_2_, MgO, etc. Catalysts operate in the 300–500 °C temperature range and at pressures ranging from atmospheric to 100 bar. Some still important drawbacks are the low catalyst resistance to coke and to sulphur from flue gas, as well as the sintering of active sites.

Thermal catalysis yields a high efficiency in terms of CO_2_ conversion and production rate and its scaling-up and final industrialization is favoured by the high flow rates it is able to withstand. There is already a fully functional commercial-scale operation e-gas plant in Werlte, Germany [67, 68]. However, the current context does not present the right conditions to ensure economically and environmentally viable production. The process entails high consumption of renewable H_2_, and this constitutes the largest contribution to the cost of the production process. Therefore, obtaining hydrogen from renewable feedstock and energy is one of the main challenges to ensure an environmentally friendly process. Here, water electrolysis from renewable energy (solar, wind, etc.) can be an economic and clean method of H_2_ production. Furthermore, although the thermodynamics of the reaction favour CO_2_ methanation at low temperatures, the high CO_2_ stability and H_2_ activation limit the reaction kinetically, and high temperatures are needed to overcome these energy barriers, which means a considerable energy input and, therefore, high operating costs for large-scale production [69–71]. Therefore, it is crucial to optimize energy efficiency and thermal control of the process to maximize a sustainable methane yield. The evolution of the energy context in the coming years, as well as the expected technological improvements, will contribute to reducing the overall cost.

Within the concept of power-to-gas, the solar approach for methane production from CO_2_ has garnered high interest. However, pure photocatalytic CO_2_ reduction using H_2_O or H_2_ still has very low yields (in the range of µmol g^−1^ h^−1^) compared to thermal catalysis due to several factors, as highlighted in Sect. 3.1. More recently, the combination of both thermal and photochemical contributions has provided additional opportunities for solar upgrading of the methanation process (Table 2). In a pioneering work, Ye et al. showed the effective application of this photothermal effect using group VIII metals for efficient CO_2_ methanation [72]. They achieved photothermal CO_2_ conversion rates several orders of magnitude (mol h^−1^ g_cat_^−1^) larger than those obtained with photocatalytic methods. Since then, different types of systems have been investigated for this purpose [38, 45, 46, 73–84]. This is indeed a burgeoning area that has driven much research. It is possible to basically distinguish between two types of systems: on the one hand, those based on noble metals, especially ruthenium and, on the other hand, systems based on transition metals, particularly nickel. Ru-based catalysts usually have higher turnover numbers, but Ni-based catalysts have received more attention mainly due to their higher availability and consequently lower cost.

**Table 2:** Some published results of photothermal CO_2_ methanation

Catalyst	Temperature (K)	Pressure (bar)	Light	Formation rate (mmol g_cat_^−1^ h^−1^)	Selectivity (%)	References
Ru/Al_2_O_3_	573	1.5	UV–Vis (Xe)	18.16 × 10^3^	99.22	[72]
Co/La_2_O_3_TiO_2_	623*	1	Vis (LED)	6	85	[82]
Ni/BaTiO_3_	598	5	UV–Vis (Xe)	103.7	> 99	[88]
Ni/BaTiO_3_	598	5	Vis (filtered Xe)	40.3	> 99	[88]
RuO_2_/Si	448	2	UV–Vis (Xe)	4.5	–	[89]
Ru/Ni_2_V_2_O_7_	350	1	UV–Vis (Xe)	114.9	99	[91]

* Except for this entry, no external heating applied

**Fig. 6:**
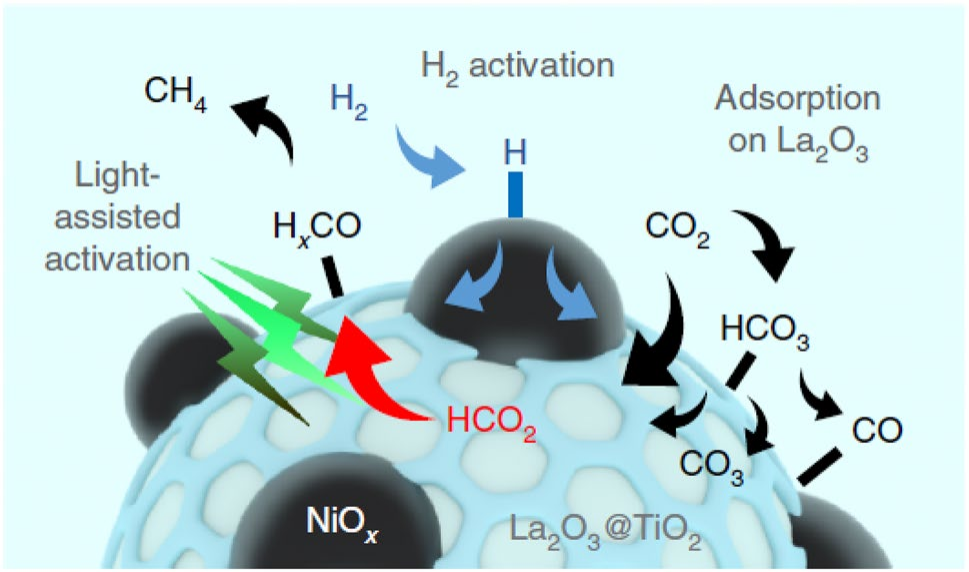
Proposed mechanism of the CO_2_ thermal-catalytic reduction pathway on NiO_x_/La_2_O_3_@TiO_2_ assisted by light. Reproduced with permission from Ref. [87]. Copyright Springer Nature 2020

For example, Amal’s group has developed extensive research on photothermally enhanced CO_2_ reduction to CH_4_ by using non-precious transition metals like Co, Cu or Ni on different metal oxide supports [82, 84–86]. The extent to which the light provides an enhancement depends on the nature of the catalyst property that is influenced by the light. For instance, in systems supported on CeO_2_, plasmonic metals like Cu drive the catalytic enhancement by the heating effect favouring the RWGS reaction. However, by using Co-based systems the improvement is not necessarily plasmonic-driven, but is attributed to the direct activation of adsorbed reaction intermediates by photogenerated hot electrons on the metal surface. Thus, under illumination, the selectivity toward CH_4_ was favoured for the Co catalyst while selectivity toward CO was promoted by the Cu catalyst. Furthermore, in the case of Co, the photogenerated hot electrons may also contribute to generate oxygen vacancies in the ceria support which act as localized charge density sites. This phenomenon was also observed using a non-plasmonic metal like Ni, coupled with CeO_2_ [86]. In addition, they showed how the incorporation of a suitable promoter with the transition metal oxide-supported catalysts can reduce the activation energies upon illumination. For instance, with La_2_O_3_ as promoter for Co/TiO_2_ catalysts, DFT calculations and in situ DRIFTS analyses revealed that the promoter facilitates CO_2_ adsorption and contributes to the photo-activation of the formate adsorbate (HCOO*) on the La_2_O_3_/titania interface, a more reactive intermediate species which further reacts with activated H* on Co NPs to produce methane. The formation of the adsorbed HCOO* intermediate species is the rate-determining step, and the enhanced photoresponse was ascribed to the promotion of this species on the La_2_O_3_ sites in Co/La_2_O_3_@TiO_2_. Furthermore, the conversion of HCOO* species could potentially increase via light activation of Co sites. They also observed a similar CO_2_ activation mechanism (Fig. 6) on NiO_x_/La_2_O_3_@TiO_2_ [87]. As noted above, CO_2_ methanation has been conventionally conducted at high temperatures where the direct CO_2_ dissociative chemisorption to CO intermediate species is the dominant route. Therefore, a photo-enhanced HCOO* activation pathway can contribute to attaining lower temperatures for process intensification.

Gascon’s group has reported another appealing case based on Ni NPs with remarkable catalytic activity using barium titanate perovskite (BTO) as a semiconductor support [88]. This titanate has received much attention in the photocatalytic water-splitting reaction. Here, Ni–BTO is a highly efficient photocatalyst to promote the complete photothermal methanation of CO_2_ without any external heating. They obtained almost 100% selectivity with a 103.7 mmol g_cat_^–1^ h^–1^ rate under UV–Vis–IR and 40.3 mmol g_cat_^−1^ h^–1^ under vis-IR irradiation. Mechanistic studies indicated that the dominant pathway reaction is non-thermal hot-electron driven while the thermal contribution to the photothermal process was minor. They ascribe their good performance to the high photochemical charge transfer efficiency of the barium titanate perovskite and its low thermal conductivity. However, the system suffers a progressive deactivation upon reuse and requires thermal reactivation.

Ozin’s group reported the use of RuO_2_ and Ru NPs supported on silicon photonic crystals for the photothermal CH_4_ production from CO_2_ and obtained conversion rates in the order of few mmol h^−1^g_cat_^−1^ under illumination with no extra-external heating [76, 89]. They showed that absorption of photons from the near-infrared (NIR) region by the silicon semiconductor contributed to the methanation enhancement by conversion of the absorbed light energy into thermal energy, then transferred to Ru centres. However, Ru light absorption across the visible–NIR region had no effect. Here, the silicon semiconductor also had a photochemical effect. DFT analysis showed that photogenerated charges could also be transferred to the Ru centres and contribute to adsorbed H_2_ activation, leading to a high density of H adatoms on the Ru surface accelerating methanation as the reaction rate is limited by the number of active surface Ru–H species. This work attributed the high photon-to-methane efficiency to the support material. However, other authors attributed the methane efficiency improvement of Ru catalysts to the LSPR of the Ru centres producing thermal and chemical effects [77, 90]. Their research also proved that the Ru response to light absorption was shape-dependent.

**Fig. 7:**
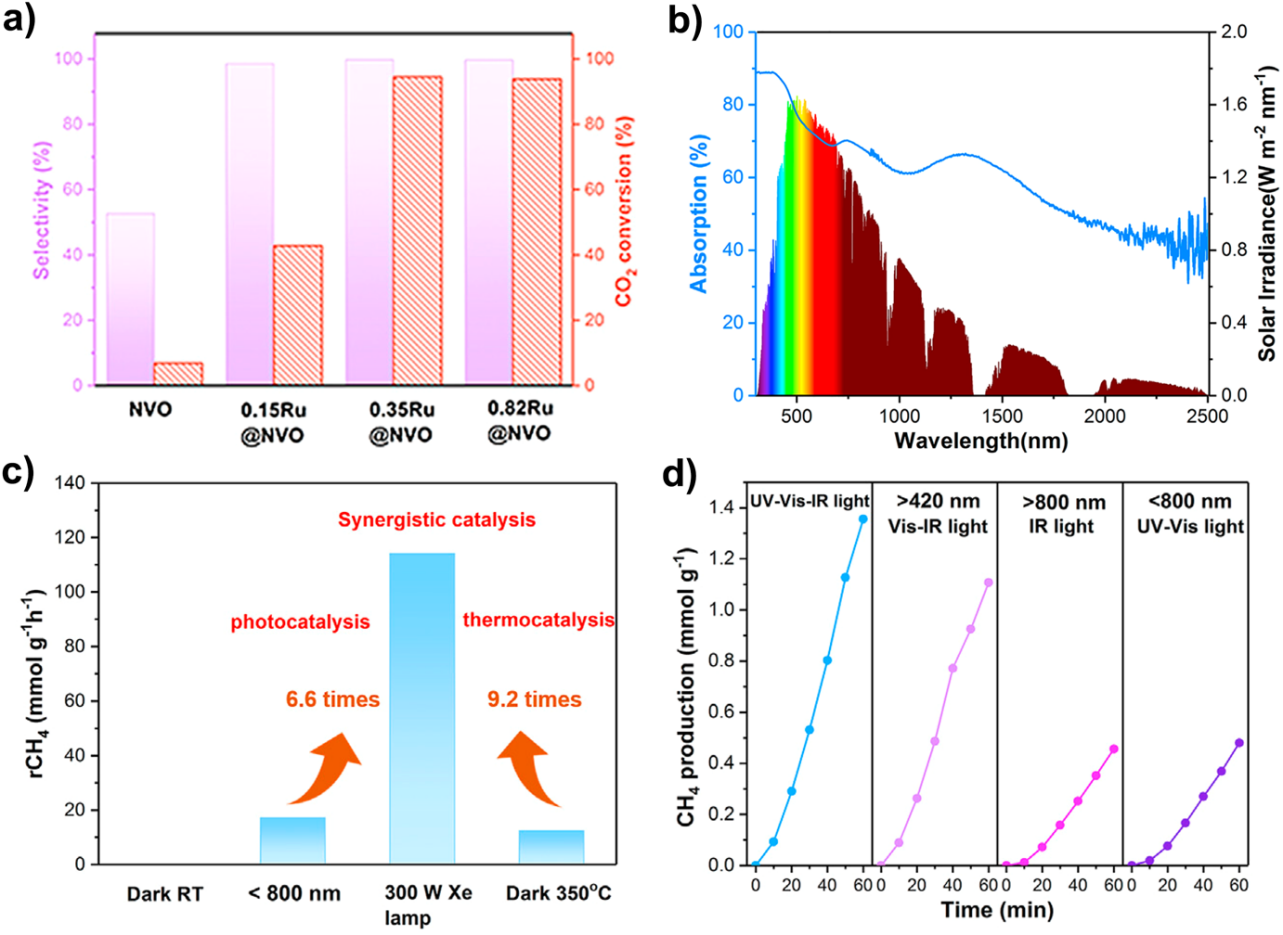
**A** CH_4_ selectivity and CO_2_ conversion; **B** UV–Vis–IR absorption spectrum of the; **C** CH_4_ production rates over 0.35Ru@Ni_2_V_2_O_7_ at room temperature in the dark, under illumination of *λ* < 800 nm, under full-Arc 300 W Xe lamp illumination, and at 350 °C in the dark and **D** CH_4_ evolution rates of 0.35Ru@ Ni_2_V_2_O_7_ as a function of time under irradiation by a full-arc 300 W Xe lamp with different filters. Reproduced-adapted with permission from Ref. [91]. Copyright Elsevier 2021

**Fig. 8:**
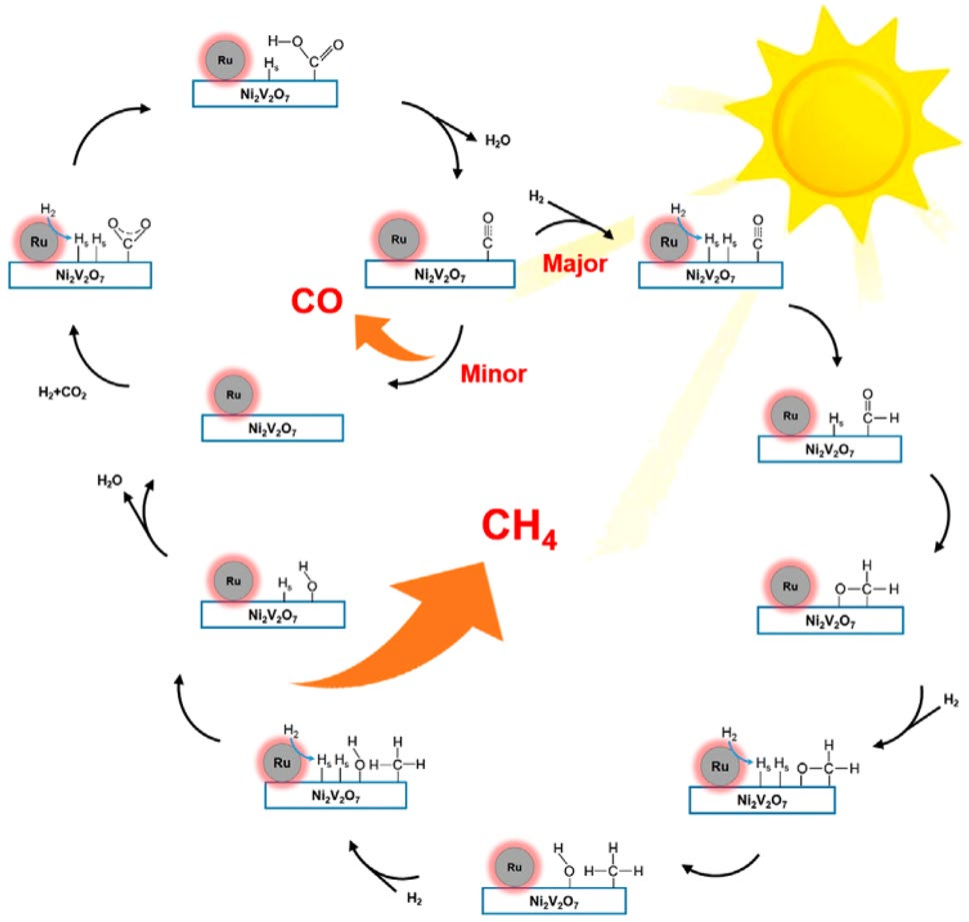
Proposed catalytic reaction mechanism for CO_2_ hydrogenation over the 0.35Ru@ Ni_2_V_2_O_7_ catalyst. Reproduced-adapted with permission from Ref. [91]. Copyright Elsevier 2021

More recently, Li et al. have reported a 0.35%Ru@Ni_2_V_2_O_7_ catalyst (Fig. 7) coupling photo/photothermal effects that uses almost full spectrum solar radiation and achieves a record rate for CO_2_ methanation: 114.9 mmol g_cat_^−1^ h^−1^ (32.8 mol g_Ru_^−1^ h^−1^ as normalized to the Ru mass), which is approximately 40 and 460 times larger than the rates obtained over the bare Ni_2_V_2_O_7_ and Ru@SiO_2_ catalysts, respectively [91]. Moreover, the system demonstrates 99% selectivity for CH_4_ production, with 93.5% CO_2_ conversion at 350 °C, approaching the thermodynamic equilibrium limit of the thermocatalytic CO_2_ methanation, by only using Xe lamp irradiation without any additional heat source. Based on in situ Fourier transform IR (FTIR) studies, DFT calculations, and temperature-programmed reduction (TPR) and temperature-programmed desorption (TPD) analyses, the authors demonstrated a cooperative mechanism coupling photocatalysis and photothermal catalysis between the Ru and Ni_2_V_2_O_7_ (Fig. 8). Upon illumination, Ru clusters act as nanoheaters increasing the local temperature. Ni_2_V_2_O_7_ also plays a role as a thermal insulator due to its low thermal conductivity favouring localized heating [92]. This allows H_2_ chemisorption and dissociation on the Ru nanoclusters. Then, H-adatoms spill over the Ru/Ni_2_V_2_O_7_ interface and diffuse onto Ni_2_V_2_O_7_, acting as a reducing species and reacting with adsorbed CO_2_ on the surface O-vacancy-rich Ni_2_V_2_O_7_. The COOH* intermediate then dissociated into CO* and H_2_O. Due to the strong interaction between the Ru/Ni_2_V_2_O_7_ interface and CO* intermediates, further hydrogenation takes place to form gaseous CH_4_. The heat released increases the temperature up to 350 °C. Ru centres act as cocatalyst and local heater under illumination. In this case, H_2_ dissociation on supported Ru sites was not the rate-limiting step. The cooperative activation routes provide an efficient mechanism for CO_2_ methanation.

### Carbon Monoxide: Reverse Water–Gas Shift Reaction

CO is a starting reagent that can be used to obtain a variety of products, from methanol to short-chain olefins and from them a plethora of additional molecules and materials [9]. Partial, 2-electron reduction of CO_2_ with hydrogen through the reverse water–gas shift (RWGS) reaction (Eq. 2 in Sect. 2.1) is therefore interesting not only for energy but also for chemical synthesis applications. As a general rule, active catalysts for RWGS are also active for methanol synthesis (see Sect. 3.4) since both reactions have common steps [14], and this is also true for photothermal catalysis (Table 3), even if catalysts of similar chemical nature can also lead the hydrogenation reaction either to carbon monoxide or to methane depending on the relative amount or the morphology of the co-catalyst [11].

**Table 3:** Some published results of photothermal reverse water–gas shift reaction

Catalyst	Temperature (K)	Pressure (bar)	Light	Formation rate (mmol g_cat_^−1^ h^−1^)	Selectivity (%)	References
In_2_O_3−x_(OH)_y_	463	1	UV–Vis (Xe)	0.15	–	[93]
Au/ZnO	873*	–	532 nm laser	4.22 × 10^–3^	95	[98]
Au/CeO_2_	673*	1	Visible (filtered Xe)	0.687	> 99	[99]
Au/TiO_2_	673	7.5	Visible (halogen)	160^§^	–	[100]
Pt/H_*x*_MoO_3-*y*_	413	1	Visible (filtered Xe)	1.2	96.5	[101]
Ni_12_P_5_/SiO_2_	473*	1.2	UV–Vis (Xe)	960	> 99	[106]

*No external heating applied

^§^CO_2_ conversion rate

A good example of the dual CO-methanol activity in photothermal catalysis is provided by the indium oxy-hydroxides developed by Ozin et al., which were initially shown to be able to produce, via RWGS, carbon monoxide at a rate of 150 μmol g_cat_^−1^ h^−1^ from diluted CO_2_ + H_2_ mixture at 190 °C and atmospheric pressure, versus 35 μmol g_cat_^−1^ h^−1^ in the dark under the same conditions, implying a photothermal enhancement factor higher than 4 [93]. In situ spectroscopic characterization and kinetic analyses, together with DFT calculations, led the authors to propose a mechanism in which a OH^−^ Lewis base next to an In^3+^ Lewis acid, together with an oxygen vacancy, assist the adsorption and heterolytic dissociation of H_2_ that permits the adsorption and reaction of CO_2_ to yield CO and H_2_O. These catalysts have been applied in an annular reactor coupled to a compound parabolic collector (CPC) under simulated sunlight and with external heating, using nickel foams as support. High selectivities to CO are reported, even reaching 100% depending on the reaction conditions [94]. Further studies of the group, however, showed that selectivity of hydrogenation over this type of catalysts is shared with methanol, as described in Sect. 3.4. Morphological control of these oxy-hydroxides has led to further enhancement of RWGS and methanol synthesis by means of an improvement of photogenerated charge carrier lifetime [95]. Ozin et al. have also recently reported the catalytic activity for RWGS of nanoporous-silica-encapsulated nickel nanocrystals photothermally heated by means of a greenhouse effect caused by the silica shell trapping the infrared radiation emitted by the nickel core upon irradiation with simulated sunlight. This system is able to convert CO_2_ at a rate of ca. 350 mmol g_Ni_^−1^ min^−1^ sustained along 10 catalytic cycles with a selectivity to CO of 90% [96].

**Fig. 9:**
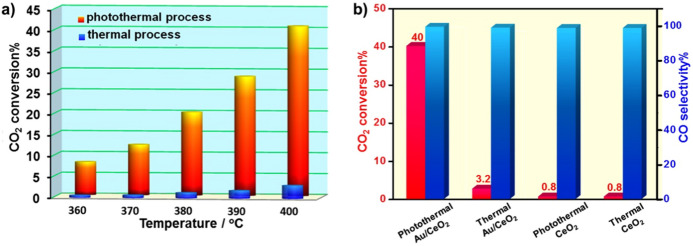
**a** CO_2_ conversion over Au/CeO_2_ in photothermal and thermal processes; **b** CO_2_ conversion and CO selectivity over Au/CeO_2_ and CeO_2_ catalysts at 400 °C under thermal and photothermal conditions. Reproduced with permission from Ref. [99]. Copyright Elsevier 2019

Regarding plasmonic or non-plasmonic metal/semiconductor systems, the nature of the metal decorating the semiconducting support does not only influence the photothermal properties of the catalytic systems, but also the selectivity towards one product or another, as in the case of photocatalysis [55] and thermal catalysis [97]. In the latter, as a general rule, metals with low CO adsorption energy tend to drive the selectivity towards this product or methanol versus the formation of methane, although modified surface reactivity of the metal, e.g. through atomic dispersion, can reverse the reaction outcome [97]. The additional factors that come into play in heterogeneous photocatalysis, like light harvesting ability or the red-ox properties of photogenerated charge carriers, however, may lead to different reactivities of supported metal nanoparticles with respect to thermal catalysis [55]. The concurrence of photonic and thermal activation mechanisms in photothermal catalysis may thus complicate the picture. For example, plasmonic gold nanoparticles supported on different oxides have been explored for photothermal CO_2_ reduction by several groups. Wang et al*.* reported the activity of ZnO modified with 20 nm sized gold particles with a plasmon resonance band centred at ca. 540 nm [98]. Raman spectroscopy was used to monitor the temperature increase induced by irradiation with a laser of 532 nm at varying intensities. The selectivity between methanation and RWGS varied as a function of the thus induced and measured temperature (up to 800 °C), the latter being favoured at higher temperatures as expected from thermodynamics. Somewhat smaller gold nanoparticles (15 nm) obtained by photodeposition were employed by Lu et al. to investigate the same reaction over a Au/CeO_2_ catalyst in which the plasmon resonance is centred at ca. 550 nm [99]. Photothermal reactions were carried out without any other heating than that provided by irradiation with a Xe lamp, with power adjusted to reach a temperature at the catalyst bed of 400 °C. In these conditions, photothermal CO_2_ conversion was ca. 10 times higher than that in pure thermal conditions at the same temperature, revealing the additional effect of light, while in the absence of Au nanoparticles, photothermal and thermal activities were essentially equal (Fig. 9). The selectivity in this case was nearly 100% towards CO, in contrast to the previously cited work that, at the same temperature and pressure, reported nearly total selectivity to CH_4_ over Au/ZnO [98]. Based on in situ DRIFTS analyses, the authors relate the improved results to the efficient hydrogen dissociation on gold nanoparticles under photothermal conditions monitored by the formation of Au-H bonds [99]. In contrast, kinetic analyses by Upadhye et al. on a Au/TiO_2_ system suggested that the LSPR increases the rate of either the hydroxyl hydrogenation or carboxyl decomposition more than any other step in the RWGS reaction [100].

In contrast to the above-described gold-based systems, platinum is a non-plasmonic metal that has been exploited in photothermal RWGS reactions. However, in the work reported by Ge et al., an oxygen vacancy-originated SPR effect was provided by the modified semiconducting support H_*x*_MoO_3−*y*_ obtained by reduction of MoO_3_ in a hydrogen flow [101]. Pt/H_*x*_MoO_3−*y*_ with sheet conformation exhibited a fourfold increase in CO production at 140 °C under visible light irradiation compared to the same reaction in dark conditions, with 96.5% CO selectivity. The role of platinum proposed by the authors is essentially catalytic for H_2_ dissociation, while the photothermal effect is provided by the SPR absorption related to the presence of oxygen vacancies and tuneable in wavelength by varying the degree of MoO_3_ reduction. In a different approach to photothermal catalysis, Zhang et al. studied the influence of external heating on the activity of Pt/TiO_2_ catalysts for RWGS [102], and concluded that thermal energy promotes the formation of hot electrons and enhances their reactivity towards adsorbed reactants, but excessively high temperature (actually that needed for thermal catalysis) suppresses the photothermal effect and essentially converts the reaction into a pure thermocatalytic one, so that the synergy of solar and thermal energies is maximum at medium temperatures, with the highest quantum efficiency for RWGS reaction attained at 400 °C.

As a highly abundant element, aluminium is an interesting alternative to noble metals for plasmonic catalysis since it presents a tuneable localized plasmon resonance covering the UV and visible regions of the spectrum [103]. Robatjazi et al. reported its reactivity combined with cuprous oxide for selective photothermal CO_2_ hydrogenation to CO under high-irradiance visible light illumination, with higher yield and selectivity than the thermal reaction at the same conditions [104]. Even if a local temperature increase is observed upon irradiation, the authors relate the reactivity to the hot electrons formed upon LSPR excitation.

A photothermal effect provoked by non-radiative electron–hole recombination in small iron oxide clusters on the surface of nitrogen-doped graphene was invoked by Szalad et al. to account for the improved reverse water gas shift activity of this catalytic system upon irradiation compared to the dark reaction at the same temperature [105]. Lifetime measurements and quenching studies by transient absorption spectroscopy led the authors to propose that Fe clusters act as recombination centres for electron–hole pairs generated upon light absorption by defective N-doped graphene and cause local heating. In contrast, a cobalt counterpart, active as thermal catalyst for RWGS, did not show the light enhancement displayed by the iron catalyst.

Another interesting class of (co)catalysts for photothermo-catalytic RWGS is metal phosphides. Taken as an archetypical example, Ozin et al. studied Ni_12_P_5_ supported on silica and formed by phosphide nanoparticles with sizes between 8 and 13 nm depending on the loading amount, and with a surface composition characterized by the formation of few-atom Ni clusters separated by P atoms [106]. In the best case, the Ni_12_P_5_/SiO_2_ catalyst gave a CO production rate of 960 mmol g_cat_^−1^ h^−1^ with a selectivity near 100% in a batch reactor under simulated concentrated (> 2 Wcm^−2^) sunlight without external heating. The photothermal effect upon absorption of light by the metal phosphide was modelled by estimating the local temperature of the catalyst surface from thermodynamic calculations based on the CO_2_ conversion at equilibrium conditions. Although less active, cobalt phosphide also gave rise to CO production in the same conditions with a selectivity over 99%.

### Methanol Synthesis

With global demand exceeding 100 million metric tons in 2021 [107], methanol is one of the main platform molecules of the chemical industry, which is used for the production of intermediates (formaldehyde, tert-butyl methyl ether, tert-amyl methyl ether and acetic acid among other compounds) for the manufacture of paints, plastics, resins, adhesives and antifreeze. In addition, methanol can also be integrated into the value chain of the petrochemical industry, for example through the methanol-to-olefin (MTO) process developed by Mobil [65, 108], which is currently gathering a growing interest due to the necessity of phasing-out fossil fuels. Likewise, the utilization of methanol derivatives such as dimethyl ether (DME), which has a cetane number (CN = 55) comparable to that of diesel, or oxymethylene ethers (OMEs), as alternative fuels is also possible.

Methanol is still mainly produced by the catalytic conversion of syngas, usually of fossil origin, over catalysts based on Cu/ZnO/Al_2_O_3_, which stand out as efficient and cost-effective formulation. Industrial methanol synthesis is thermally activated and it takes place at high pressures (50–250 bar) and moderate temperatures (200–350 °C) [65, 109]. The direct synthesis of methanol from pure CO_2_/H_2_ mixtures remains a challenge, but due to the requirements of reducing the carbon footprint, this reaction is attracting growing scientific interest. Recently, very promising results have been obtained with a Co/In_2_O_3_ under thermal conditions operating at 300 °C and 50 bars [110].

In the present energy context, the photothermal hydrogenation of CO_2_ to methanol is particularly relevant [10], and it has already been approached in a few works (Table 4). In this way, the classical formulation of the methanol synthesis catalyst, Cu/ZnO, has been tested under photothermal conditions [111]. Operating at atmospheric pressure with a H_2_/CO_2_ ratio of 3 and at 220 °C, illumination with visible light enables an increase in methanol production by 1.5 times to reach 132 µmol g^−1^ h^−1^. Linear correlation of activity with irradiance ranging from 0 to 0.6 W cm^−2^ is found. However, the selectivity towards CO, which generation is also promoted by illumination, is much higher (ca. 90%) under these low-pressure conditions. Activation by light in this catalytic system has been related to the generation of hot electrons by localized surface plasmon resonance in the Cu particles. Likewise, using the classical industrial catalyst for methanol synthesis, Cu/ZnO/Al_2_O_3_, a very high photothermal yield of methanol (ca.7.8 mmol g_cat_^−1^ h^−1^) is obtained using a total pressure of 21 bar at 225 °C under illumination (irradiance 600 mW cm^−2^ in the 350–800 nm range). This represents a 32% increase in methanol production relative to pure thermal conditions, without any significant variation in selectivity. This promotion by simulated solar light is attributed to the excitation of both the ZnO bandgap and the Cu plasmon, on the basis of experimental and computational studies by DRIFT and DFT, respectively [112]. Similarly, Pd/ZnO catalyst has been used for photothermal production of methanol [113]. Using a feed with H_2_/CO_2_ of 3, a total pressure of 12 bar and a temperature in the 190–270 °C range, this catalyst also shows a clear increase in methanol activity of 1.5–3.0 times under irradiation with a 500 W Hg lamp. In addition, CO production rises by 1.6–4.7 times, depending on the temperature. In this regard, it appears that at high temperatures (250–270 °C), irradiation promotes RWGS to a larger extent than methanol synthesis, which reaches an activity of 4 mmol g^−1^ h^−1^. Experimentally, it is found that for this catalyst, the plasmon of the Pd nanoparticles is red-shifted to 570 nm due to interaction with ZnO, allowing activation under visible light.

**Table 4:** Some published results of photothermal methanol synthesis

Catalyst	Temperature (K)	Pressure (bar)	Light	Formation rate (mmol g_cat_^−1^ h^−1^)	Selectivity (%)	References
Cu/ZnO	493	1	Visible (filtered Xe)	0.13	10	[111]
Cu/ZnO/Al_2_O_3_	498	21	UV–Vis (Xe)	7.8	80	[112]
Pd/ZnO	523	12	UV (Hg)	3.8	51	[113]
Pt-Au/ZIF8	423	32	UV–Vis (Xe)	3 mmol/h^§^	96	[114]
Co/TiO_2_	393	1.3	UV–Vis (Xe)	0.0396	99.9	[115]
Bi_x_In_2−x_O_3_	503	1	UV–Vis (Xe)	0.158	15	[117]

^§^Amount of catalyst not indicated

An interesting promotion of the photothermal methanol production was reported using Pt nanocubes and Au nanocages embedded into a zeolitic imidazolate framework (ZIF-8) [114]. This nanoarchitecture allows selective heating of Au structures to activate Pt sites, while the metal–organic framework (MOF) structure contributes to thermally insulating these metallic centres. This catalyst shows a remarkable enhancement in selectivity towards methanol production, with only a minor amount of formic acid formation, working in aqueous solution in a batch reactor at 150 °C and 32 bar (with H_2_/CO_2_ of 3). Under similar reaction conditions, using an aqueous suspension, a catalyst based on CoO and Co^0^ dispersed on TiO_2_ was applied to the photothermal production of methanol [115]. In this system, metallic particles electronically connect the two oxide semiconductors in a z-scheme. Catalytic activity tests were performed using a batch reactor at 120–140 °C with an autogenous pressure ratio of H_2_/CO_2_of 2.5. Under illumination with a Xe lamp, this catalyst generates up to 46.5 μmol g^−1^ h^−1^ methanol, with some traces of methane.

**Fig. 10:**
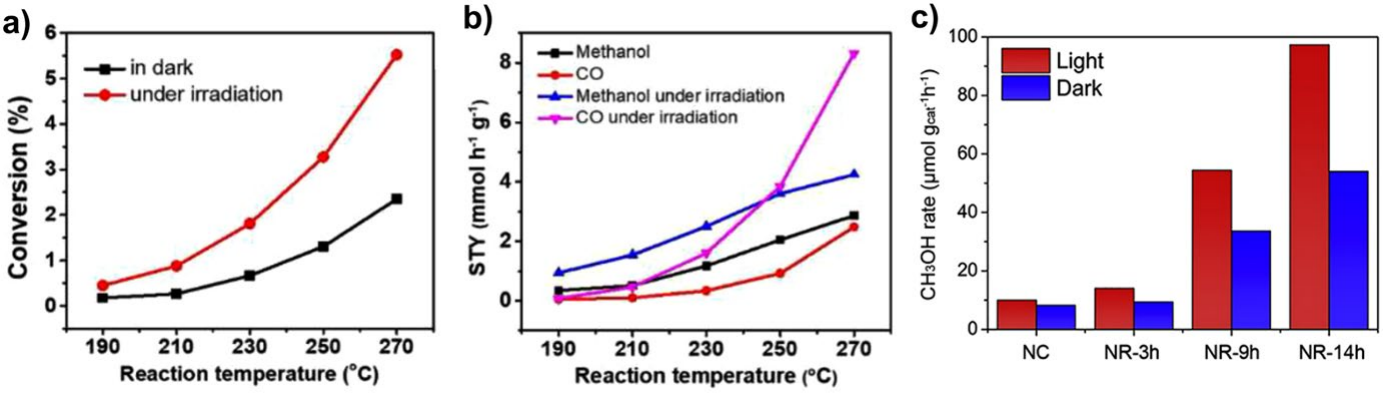
**a** Conversion of CO_2_ and **b** selectivity towards methanol and CO as a function of temperature for the CO_2_ hydrogenation over Pd/ZnO catalyst with and without light irradiation and **c** methanol rate of different the In_2_O_3−x_(OH)_y_ nanocrystalline catalysts during hydrogenation of CO_2_ at 250 °C with and without solar irradiation. Reproduced with permission from Ref. [116]. Copyright Elsevier 2018

Efficient photothermal methanol production over metal-free catalysts based on In_2_O_3−x_(OH)_y_ nanostructures can be at achieved at atmospheric pressure and working at temperatures in the 200–300 °C range, as summarized in Fig. 10 [116]. With this system, illumination almost doubles the production of methanol, achieving a maximum rate of 0.06 mmol g^−1^ h^−1^ and a selectivity of about 53%, with CO as the other reaction product. This catalyst proved to be stable for at least 20 h on stream. The high performance of these catalysts has been attributed to the existence of frustrated Lewis pairs on the surface. These surface centres are formed by a surface hydroxide, In-OH, in the vicinity of an oxygen vacancy, In-O_v_, and they are very active for H_2_ activation. Those types of centres have been reported to be present on Bi_x_In_2-x_O_3_ materials, which have also shown to be efficient photothermal catalysts [117]. However, the Bi-doped oxide is three orders of magnitude more photoactive for the reverse water gas shift reaction than unmodified In_2_O_3_. In addition, this catalyst also presents high activity towards methanol production, with a rate of 158 μmol g^−1^ h^−1^ at 230 °C under visible illumination. In addition to modulating selectivity, Bi incorporation in the In_2_O_3_ results in a shift of the light absorption towards the visible range, which can be beneficial for photoactivation using solar light. In a similar way, black indium oxide, H_z_In_2_O_3−*x*_(OH)_*y*_, which is synthesized by mild reduction and contains oxygen vacancies, hydroxyls, and hydride species, also shows promising photothermal catalytic activity for methanol production [118]. This catalyst can work in tandem, by first producing CO and subsequently methanol, making it possible to reduce the hydrogen concentration in the feed and operate at ambient pressure. Then, using a H_2_:CO_2_ ratio of 3:1 at 250 °C the initial methanol rate reached 14.92 µmol g^−1^ h^−1^ with a selectivity of 32.6%, although following 75 h on stream the rate decreased to 7.54 µmol g^−1^ h^−1^ while the selectivity increased to 49.23%.

Interestingly, production of higher alcohols such as ethanol from hydrogenation of CO_2_ under photothermal conditions has also been reported recently. In this respect, using a batch reactor pressurized at 2.8 bar (H_2_/CO_2_ ratio of 5) at 235 °C and loaded with Co@C nanoparticles promoted with Na resulted in selectivity of 6.5% for ethanol, although methane (50.2%) and larger hydrocarbons (38.5%) were the major products, and CO was also produced (4.8%) [119].

## Conclusions and Outlook

Light-heat synergies open up interesting possibilities with a view to improving both photocatalytic and thermocatalytic reactions for CO_2_ conversion, the former by increasing the still-low reaction rates attained, the latter by smoothing the necessary operation conditions and opening new reaction pathways that may lead to different selectivities. In this article, we have tried to provide an updated overview of the known photothermal catalysis foundations and the most prominent examples of photothermal CO_2_ conversion, with special emphasis on the most interesting reaction products and the selectivities towards them that can be attained.

In view of the published works, the first conclusion that can be drawn is that photothermal catalysis research moves towards the use of hydrogen as reducing agent rather than the artificial photosynthesis approach that uses water as electron donor. In this sense, the main challenges may lie on diminishing reaction temperature as to favourably compete with the relatively highly developed thermocatalytic CO_2_ hydrogenation processes, as well as to avoid the deactivation of catalysts the latter suffer from due to coke formation or catalyst sintering.

Reaction mechanisms and photonic-thermal synergy pathways are still quite unclear and, from the reaction route point of view, it can be said that photothermal catalytic CO_2_ reduction processes are still in their infancy. What seems clear, however, is that the term “photothermal catalysis” encompasses a large number of different mechanisms and catalytic systems, and that the beneficial effect arises from a combination of factors, among which the dominance of one or another, which may actually determine the reaction outcome, depends highly on the nature of the (photo)catalytic material as well as on the operation conditions. In this respect, widening the in situ and operando spectroscopic studies, including spatially and temporally resolved temperature measurements (direct or indirect) will probably play a key role in the near future.

## Data Availability

This article is a literature revision and does not contain any original data that could be made available.
